# Impact of opening hermetic storage bags on grain quality, fungal growth and aflatoxin accumulation

**DOI:** 10.1016/j.jspr.2016.10.003

**Published:** 2016-10

**Authors:** Timothy Tubbs, Dieudonne Baributsa, Charles Woloshuk

**Affiliations:** a Department of Botany and Plant Pathology, Purdue University, West Lafayette, IN 47907, USA; b Department of Entomology, Purdue University, West Lafayette, IN 47907, USA

## Abstract

Purdue Improved Crop Storage (PICS) bags are used by farmers in Sub-Saharan Africa for pest management of stored grains and products, including maize. These bags hermetically seal the products, preventing exchange with external moisture and gases. Biological respiration within the bags create an environment that is unsuitable for insect development and fungal growth. This study was conducted to determine the impact of routine opening of the storage bags for maize consumption on fungal growth and aflatoxin contamination. Maize with moisture contents (MC) high enough to support fungal growth (15%, 16%, 18% and 20%) was stored in PICS bags, which were opened weekly and exposed to humid conditions (85% RH) for 30 min over a period of 8 weeks and 24 weeks. Monitors indicated that oxygen defused into the open bags but did not reach equilibrium with the bottom layers of grain during the 30-min exposure period. Fungal colony forming units obtained from the grain surface increased 3-fold (at 15% MC) to 10,000-fold (at 20% MC) after 8 weeks. At both 8 weeks and 24 weeks, aflatoxin was detected in at least one bag at each grain moisture, suggesting that aflatoxin contamination spread from a planted source of *A*. *flavus*-colonized grain to non-inoculated grain. The results indicate that repeatedly breaking the hermetic seal of the PICS bags will increase fungal growth and the risk of aflatoxin contamination, especially in maize stored at high moisture content. This work also further demonstrates that maize should be properly dried prior to storage in PICS bags.

## Highlights

• The effect of routine opening of hermetically stored maize is examined.
• With high moisture grain, there is an increasing risk of fungal growth and aflatoxin accumulation.
• Reduction in seed viability decreases with grain moisture and time in storage.

## 1 Introduction

Purdue Improved Crop Storage (PICS) is a trademarked hermetic bag system that consists of two inner bags made of high-density polypropylene with an outer woven polypropylene bag. Originally developed to reduce insects damage to stored cowpeas in western and central Africa (Murdock et al., 2012), the bags have been evaluated for other commodities such as maize, groundnut, and pigeon pea (Baoua et al., 2014, Murdock et al., 2012, Sudini et al., 2015, Vales et al., 2014, Williams et al., 2014). Biological activity within the bags, including respiration from the stored products and/or the insects and grain, decreases oxygen and increases carbon dioxide to levels that are insecticidal. Results from these studies indicate that PICS bags provide an affordable management solution that preserves grain quality and reduces dependence on postharvest insecticide applications.

With increased adoption of PICS and other hermetic bags, efforts have focused on applying the technology in countries of Sub-Saharan Africa. Many areas in this region have rainy seasons that often coincide with the harvest, drying and storage period. In these areas, the major questions concern the growth of mycotoxigenic fungi, especially in stored maize and groundnuts. Williams et al. (2014) addressed some of these questions in a study of maize with different moisture contents (12%, 15%, 18%, and 21%) stored in PICS bags and woven bags. They found that moisture content (MC) of grain stored in the woven bags was influenced by the surrounding storage environment and aflatoxin content was higher in the bags with higher MC. In contrast, PICS bags maintained the original MC of the grain for two months of storage, and prevented accumulation of aflatoxin, even after two months.

Subsistence farmers routinely remove grain from their storage bags for consumption or sale. A study implemented in Niger on the impact of the frequency of opening PICS bags found no detrimental effect on insect control (Baoua et al., 2013). However, no study has examined the effect of opening frequency on fungal growth and aflatoxin accumulation in moist grain. Frequent opening eliminates the hermetic seal of PICS bags that is essential for the efficacy of this technology in maintaining grain quality. In this current study, we measured changes in fungal growth, aflatoxin accumulation, seed germination, and grain moisture of stored moist maize caused by routinely opening PICS bags under humid conditions. Our results provide insight into how such activity affects grain quality.

## 2 Methods

### 2.1 Experimental design

#### 2.1.1 Maize conditioning

Maize used in this study was produced in 2014 at the Agronomy Center for Research and Education (Purdue University West Lafayette, IN). The grain was placed in four plastic drums (113.6 L), each containing 40 kg of maize seed. The initial grain was 15% MC and three drums were conditioned to 16%, 18% and 20% MC by adding the appropriate amount of water, mixing the grain for 3 h on a drum roller (Morse Manufacturing Co., East Syracuse, NY), and incubating the sealed-drums for 7 days at 4 °C. The final MC of the conditioned grain were 15% (14.95 ± 0.03), 16% (16.35 ± 0.02), 18% (18.51 ± 0.07) and 20% (20.13 ± 0.04).

The four grain moistures were replicated seven times (total of 28 hermetic bags). Beginning at 8 weeks after initial storage, three replicate-bags for each grain moisture were opened every 7 days and placed in a humidity chamber (85% RH) for 30 min, then re-sealed and returned to the original storage conditions. Another set of three replicate-bags for each grain moisture was treated the same for 24 weeks. One bag for each grain moisture remained sealed for the entire 24 weeks.

#### 2.1.2 Hermetic bags

**Fig. 1:**
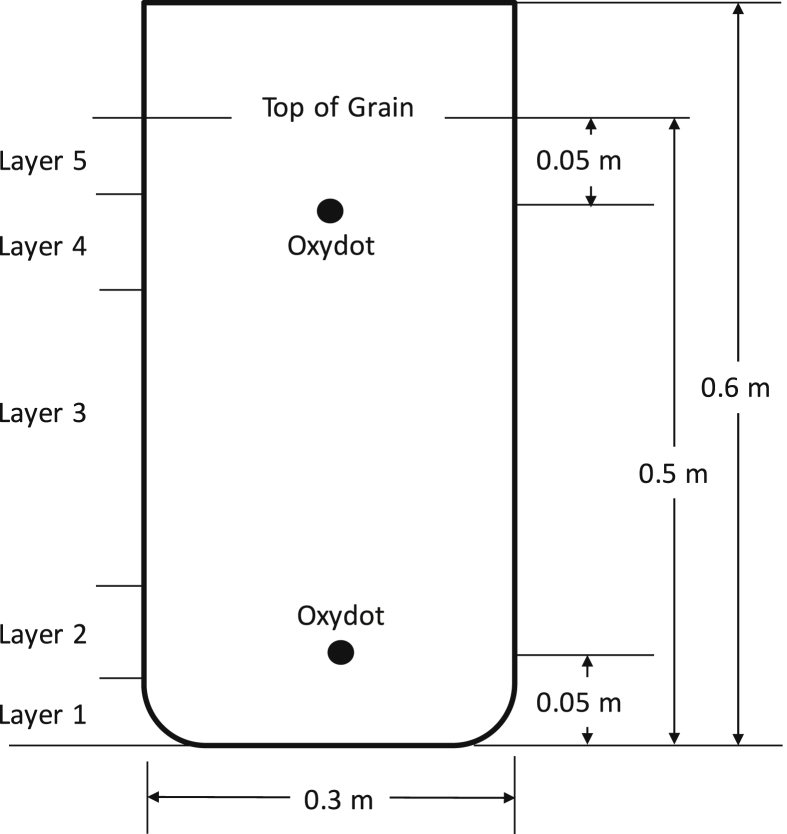
Diagram of the modified PICS bag. Layers consisted of: (Layer 1) 1 kg of conditioned maize; (Layer 2) three small mesh bags containing *A*. *flavus*-colonized maize and three small mesh bags containing non-colonized maize; (Layer 3) 3 kg of conditioned maize; (Layer 4) three small mesh bags containing *A*. *flavus*-colonized maize and three small mesh bags containing non-colonized maize; and (Layer 5) 1 kg of conditioned maize.

Three-layer hermetic bags (0.3 m by 0.6 m) were constructed from 50 kg PICS bags. The bottom and sides of the bags were sealed with an electrical heat sealer (Uline H-86 Impulse Foot Sealer). For monitoring oxygen levels, an Oxydot (OxySense Inc, Dallas, TX) was attached to a plastic Petri dish (100 mm), which was then affixed to the inner bag with tape. One oxygen monitor was placed approximately 50 mm from the bottom of the bag and another about 50 mm below the grain surface near the top of the bag (Fig. 1). The Oxydot monitors were visible through the bag layers.

Each hermetic bag was filled with the following layers from bottom to top (Fig. 1): (Layer 1) 1 kg of conditioned maize; (Layer 2) three small mesh bags containing a 100 g of *A*. *flavus*-colonized maize each and three small mesh bags containing a 100 g of non-colonized maize each; (Layer 3) 3 kg of conditioned maize; (Layer 4) three small mesh bags containing 100 g of *A*. *flavus*-colonized maize each and three small mesh bags containing a 100 g of non-colonized maize each; and (Layer 5) 1 kg of conditioned maize. Two bags from each treatment group contained temperature/humidity data loggers (Lascar Electronics Inc., Erie, PA) placed within Layers 2 and 4. These data loggers were programmed to record measurements every 20 min. The top of each bag liner was twisted and folded to form an airtight seal and secured with a plastic zip-tie. Bags were stored at 25 °C.

#### 2.1.3 *Aspergillus flavus*-colonized maize

*Aspergillus flavus* NRRL 3357 was grown for 7 days on Petri dishes (100 mm × 15 mm) containing potato dextrose agar (PDA) medium. After soaking 25 kg of maize in water overnight, the grain was distributed into 10 mushroom spawning bags (Gourmet Mushroom Supply) and autoclaved twice. Each bag of grain was inoculated with one culture dish of the fungus, which was diced into small pieces. Bags were incubated at 25° C and mixed several times each day to assure uniform colonization. After 3 days, the colonized maize was removed from the bags, rinsed twice with water to remove conidia, dried for 24 h by spreading the grain on a greenhouse table, and then placed in an oven (38° C) until the moisture content reached 15%. Small mesh bags were prepared, containing 100 g of the colonized maize. Non-colonized Control bags contained grain with 15% moisture.

### 2.2 Data collection

#### 2.2.1 Oxygen concentration

Oxygen concentration in the bags was measured with an OxySense 5250i oxygen reader (OxySense Inc) before opening and after re-sealing. After 8 and 24 weeks of treatment, grain was collected from the top, middle and bottom layers of three replicated bags for moisture and germination analyses.

#### 2.2.2 Grain moisture and seed germination

Moisture was measured according to ASAE standard protocols (Standards, 2012). Dry weight was determined on 15 g of maize after 72 h in a 103° C oven. For each sample collected, at least three replicates were measured. Germination was determined on 150 kernels (3 replicates of 50 kernels) as described by Williams et al. (2014), except the incubation temperature was 22° C.

#### 2.2.3 Surface-borne fungi

To determine the number of fungi propagules on the grain surface prior to starting the experiment, three samples were removed from each barrel of conditioned maize. After storage treatments, a total of nine samples were collected from each bag (three each from the top, middle and bottom layers). For each sample, 40 g of maize was placed in a flask containing 100 mL of sterile 0.05% Triton X-100 solution, and mixed for 2 min. The wash was serially diluted and plated onto malt salt agar (MSA) medium (McDonough et al., 2011) and PDA medium. Colony numbers were determined after 3–5 days of incubation at 30° C, and data for each bag were combined for analysis.

#### 2.2.4 Aflatoxin analysis

Aflatoxin concentrations in the small bags containing *A*. *flavus*-inoculated and non-inoculated grain were determined. The three bags of *A*. *flavus*-inoculated grain at the top of the hermetic bag (Layer 4, Fig. 1) were combined and mixed thoroughly. Approximately 40–100 g of the combined maize was ground in a Bunn G3 HD coffee grinder (Bunn, Springfield, IL). The bags of *A*. *flavus*-inoculated grain in Layer 2 and the non-inoculated grain in Layers 2 and 4 (Fig. 1) were processed similarly. From each sample, three subsamples (0.5 g) of the ground maize were extracted by shaking overnight in 2 ml of acetonitrile. Extracts were centrifuged and the supernatant was filtered through a 0.56 μm filter (Supelco, Bellefonte, PA). Samples were analyzed by HPLC as described by Williams et al. (2014). Aflatoxin B1 was quantified by comparing peak areas with B1 standards (Sigma Chemical Co., St. Louis, MO).

### 2.3 Statistical analysis

SAS 9.4 (SAS Institute Inc., Cary, NC) with PROC GLM and CONTRAST was used to analyze data and determine statistical significance.

## 3 Results

### 3.1 Oxygen concentration

**Fig. 2:**
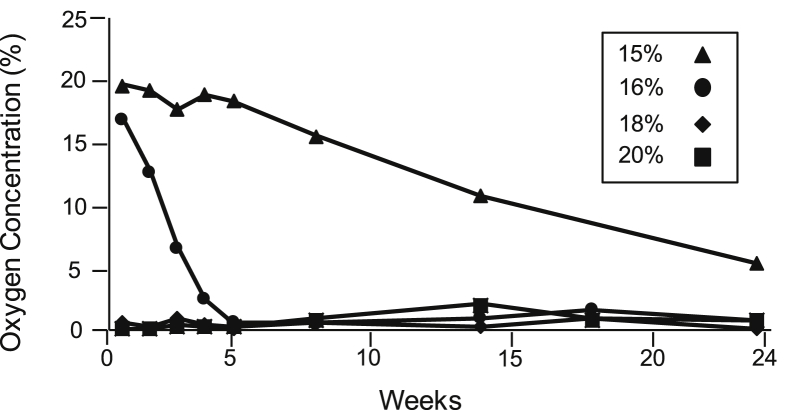
Changes in oxygen concentration in the PICS bags sealed for 24 weeks containing maize at 15%, 16%, 18% and 20% moisture content.

Oxygen concentration was influenced by maize grain moisture. Within the first week of storage, the oxygen concentration decreased to near zero at grain moistures of 18% and 20% MC (Fig. 2). For the maize at lower grain moisture, the oxygen concentration decreased more slowly. Bags containing 16% MC maize reached zero oxygen concentration after 5 weeks, and the grain at 15% MC reached 15.5% O_2_ after 8 weeks and 5% O_2_ after 24 weeks.

**Fig. 3:**
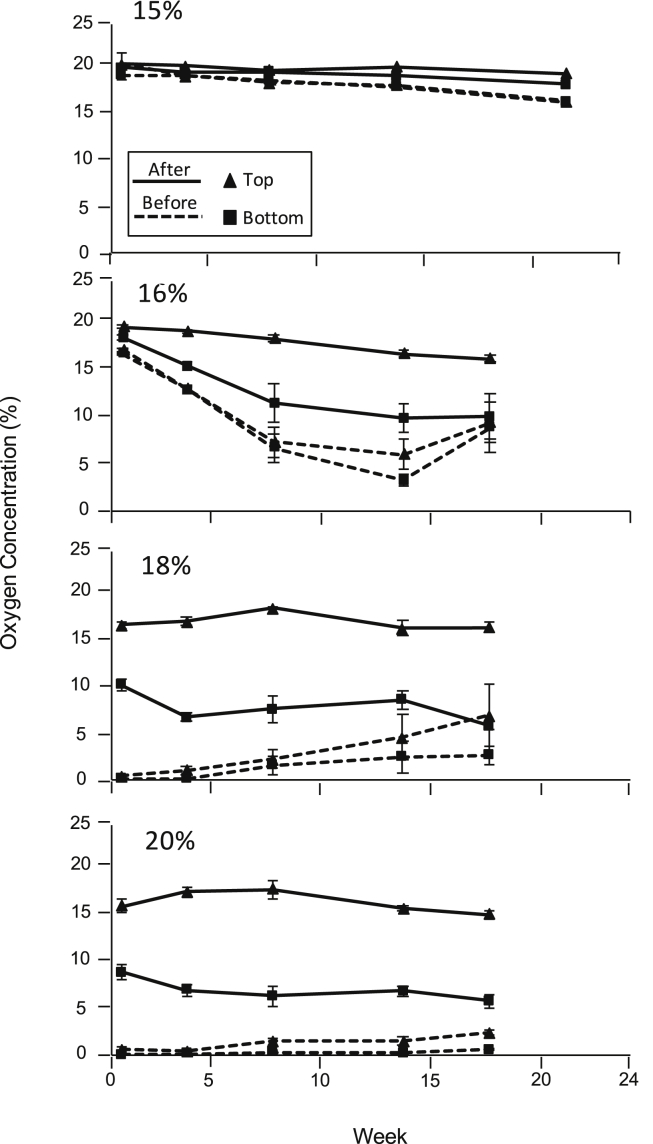
Effect of opening PICS bags on oxygen concentration for maize at 15%, 16%, 18% and 20% moisture content. Measurements were taken from the top and bottom of the bags before opening and after resealing. Data are mean values of 3 replicates per moisture with standard error bars.

In the hermetic bags sealed for the entire 24-week experiment, minimal difference in the oxygen concentrations was detected between the top and bottom of the bags. Oxygen concentrations at the top and bottom of the hermetic bags were measured weekly just before opening and immediately after resealing. After opening the bags for 30 min, the oxygen concentration at the top of the bags increased with the diffusion of oxygen from the outside atmosphere (Fig. 3). For all grain moistures, outside oxygen defused to the bottom of the bags, although 30 min was insufficient to reach equilibrium with the top layer. Opening the bags also impacted the reestablishment of reduced oxygen levels after reclosing. In contrast to the bag sealed for 24 weeks, the 16% MC grain did not reach zero O_2_ concentration. At 8 weeks, the oxygen concentration was 7.2% prior to opening the bag and remained between 7% and 11% until the end of the experiment. Even in the bags containing the higher moisture grain, the oxygen concentration failed to return to zero. At 8 weeks, the oxygen concentrations were 1% and 2% in the bags containing maize at 20% and 18% moisture, respectively, and did not change during the remainder of the experiment.

### 3.2 Relative humidity

Grain temperatures at the top and bottom of the bags were essentially identical (data not shown). Humidity in the bags sealed for 24 weeks remained steady throughout the experiment, with readings at the bottom of the 15% and 16% MC bags consistently 2%–3% higher at the top than the bottom. In the sealed bag containing 15% MC grain, the average humidity (top and bottom) was 78% RH at 8 and 24 weeks. For 16%, 18%, 20% MC treatments, the average RH at 8 weeks were 82%, 88%, and 92%, respectively. At 24 weeks the RH values were essentially unchanged with a slight increase of 1%, 3% and 2%, respectively. In bags that were opened weekly, the RH increased over time in the bags with the higher moisture grain. In the bag containing 16% MC grain, the RH was 82% at 8 weeks and increased to 86% at 24 weeks. In the bag containing grain at 18% MC, the RH was 87% at 8 weeks. The bottom layer registered 95% RH at 24-weeks. The data logger at the top of the bag malfunctioned. Similarly, the RH in the bag containing 20% MC maize increased from 92% at 8 weeks to 97% at 24 weeks. In bags containing grain with the lowest moisture (15% MC), the RH was 78% at both 8 and 24 weeks.

### 3.3 Grain moisture

**Fig. 4:**
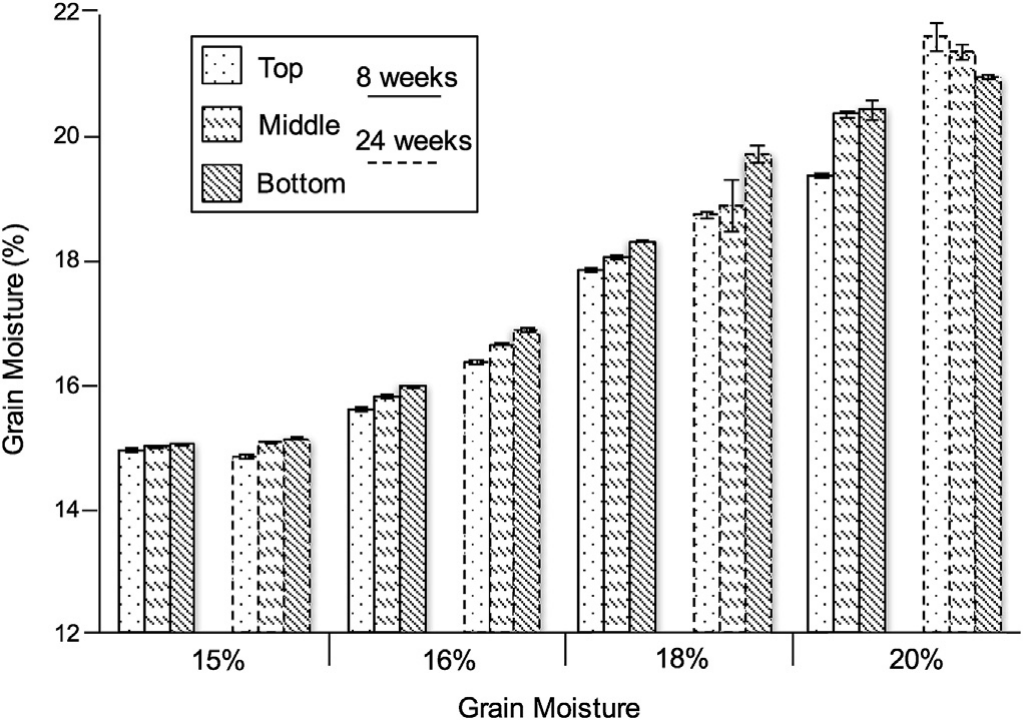
Effect of weekly opening of PICS bags on grain moisture. After 8 weeks and 24 weeks, 3 samples were collected from the top, middle, and bottom layers of PICS bags containing maize at 15%, 16%, 18% and 20% moisture content. Data are mean values of the 3 replicate bags with standard error bars.

We also measured grain moisture after 8 and 24 weeks in the bags that were opened weekly and at 24 weeks in the set of sealed bags. Moisture of maize sampled from the bottom of the opened bags was generally higher than the samples from the top (Fig. 4). When we combined the moisture values obtained three layers in the bags, the mean moisture contents were 15.02%, 15.81%, 18.11%, and 20.10% after 8 weeks for the 15%, 16%, 18%, 20% treatments, respectively. Only the value for the 16% MC grain was significantly different (P < 0.05) from the initial starting moistures. After 24 weeks, the 15% MC treatment did not change significantly. However, moisture content of grain stored at 16%, 18%, and 20% increased significantly to 16.66%, 19.16% and 21.36%, respectively. In the one bag for each moisture treatment that was sealed for 24 weeks, the moisture content changed very little. The average grain moisture (±SE) in these bags after 24 weeks was 15.1 ± 0.04, 16.44 ± 0.05, 18.59 ± 0.04, and 20.04 ± 0.1.

### 3.4 Seed germination

The initial germination rate of the grain ranged from 96 to 98.7% (Table 1). Increasing moisture content had a detrimental effect on germination. Only the maize at 15% MC maintained the initial germination rate during 8 weeks of storage. However, after 24 weeks, the germination rate for this treatment was reduced by nearly 80%, which was half the germination rate of the grain in the bag sealed for 24 weeks. Whether sealed or opened weekly, the ability to germinate was essentially lost when grain was stored for 24 weeks at the higher moistures (18% and 20% MC).

### 3.5 Surface fungi

**Fig. 5:**
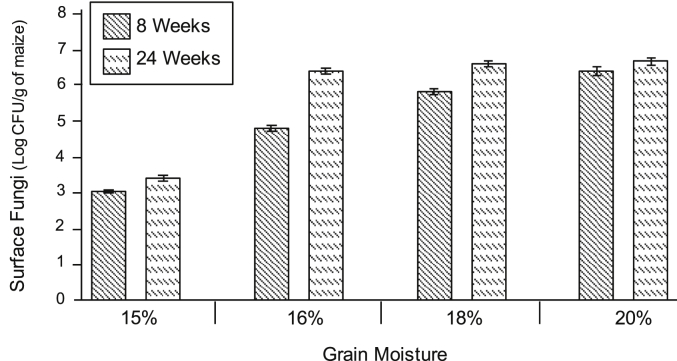
Effect of weekly opening of PICS bags on fungal growth. After 8 weeks and 24 weeks, the 6 small bags containing non-inoculated maize (3 at top and 3 at bottom) in each PICS bag were collected. A sample (45 g) from each was washed to obtain surface fungi, and the number of fungi propagules was determined by serial dilution and plate counts. Data are presented as the combined means of the 3 replicate PICS bags for each moisture treatment with standard error bars.

Washes taken from the initial grain yielded 6.6 ± 0.7 × 10^2^ colonies per gram of maize. In the storage bags opened weekly, fungal counts in the grain stored at 15% MC increased 0.5 log units after 8 weeks and 0.9 log units at 24 weeks (Fig. 5). In the bags containing the higher moisture grain (16%, 18% and 20%), fungal counts after 8 weeks increased 2.4, 3.5, and 4.1 log units, respectively. After 24 weeks, the fungal counts were 4.1, 4.3 and 4.4 log units greater than in the initial grain, respectively,. Although not quantified, *Fusarium* species were more prevalent at 8 weeks and 24 weeks in the grain with 15% MC than in the grain with higher moisture contents. *Penicillium* spp. along with *Aspergillus* spp. dominated in the grain at 16% and 18% MC. At 24 weeks, yeasts were identified in washes from grain at 18% and 20% MC, with higher numbers in the grain samples at 20% MC.

### 3.6 Aflatoxin

The small bags of *A*. *flavus*-colonized maize placed at the top and bottom of each hermetic bag contained high quantities of aflatoxin B1 (AFB1) (7115.5 ± 1813.6 ng AFB1/g of maize). The concentration did not increase appreciably in any of the treatments, (data not shown). In the bags sealed for 24 weeks, 54 ng AFB1/g was measured in non-inoculated grain in the bag containing the 20% MC but was not detected in the surrounding maize. We also detected aflatoxin in non-inoculated maize in the bags that were opened weekly (Table 2). At 8 weeks, aflatoxin was detected in one bag of each of the moisture treatments, with the highest amount in the 18% and 20% MC grain. By 24 weeks, the non-inoculated maize in all three bags of the 16% MC treatment contained aflatoxin. Two bags of the 18% and 20% MC treatments also contained aflatoxin (Table 2).

## 4 Discussion

Prevention of *A*. *flavus* growth and aflatoxin accumulation in maize can be achieved by drying the grain to below 14% (a_w_ < 0.70) (Magan and Aldred, 2007). At this range of moisture content, only the more xerophilic fungi will grow (Lacey, 1989). Previous studies have shown that sealing grain in a hermetic bag, such as PICS, will prevent changes in grain moisture (Mutungi et al., 2014, Njoroge et al., 2014, Williams et al., 2014). In this current study, we also measured very little change in grain moisture after 24 weeks of storage in the PICS bags that remained sealed. Our results indicate that opening the bags weekly for eight weeks did not affect moisture content of the grain. However, the grain with higher moisture contents (16%, 18%, and 20%) tended to increase approximately 1% moisture during the 24 weeks. These results suggest that routine opening will not result in a significant increase in grain moisture, especially if the grain is dried to at least 15% MC.

Murdock et al. (2012) described the control of weevil populations in cowpeas by storage in PICS bags. The efficacy of this technology relies on the depletion of oxygen and the increase in carbon dioxide caused by insect respiration (Murdock and Baoua, 2014). With dry grain and low insect populations inside the bags, Njoroge et al. (2014) found that it took over two months for the oxygen concentration to reach 5%, the level at which larval feeding activity is impaired (Murdock et al., 2012). Williams et al. (2014) found that oxygen concentration in 12% maize stored in PICS bags remained above 10% after two months, but at 15% MC the oxygen concentration was near zero after one month. In our experiment, the oxygen concentration in the sealed bag containing maize at 15% MC declined to approximately 6% after 21 weeks and 5.3% at 24 weeks. The difference observed between the two studies is likely related to the quality of maize used by Williams et al. (2014), which had a germination rate of only 40% compared to the 97% germination rate in our study. The lower germination rate suggests that the grain contained higher populations of storage fungi, which are able to grow at 15% MC, thereby reducing the oxygen content at a faster rate.

We discovered that an oxygen gradient formed when the hermetic seal of the PICS bag was broken. Once the bags are opened, external oxygen quickly diffuses into the top layer of the bags. The gases diffuse toward the bottom of the bag but fail to reach equilibrium within the 30 min exposure period. Previous research has indicated that the grain porosity, moisture and temperature influence the diffusion rate of oxygen and carbon dioxide through grain (Bailey, 1959, Huang et al., 2013, Shunmugam et al., 2005). Our results indicate that the grain with higher moisture contents (18% and 20%) did not reach equilibrium with the oxygen in the external air compared to grain at the lowest moisture content (15%). This apparent decrease in diffusion rate through the top layers may be due to grain moisture; however, this explanation does not account for potential loss of oxygen because of higher respiration rate of the wetter grain (Huang et al., 2013). A higher respiration rate was also responsible for the decrease in oxygen concentration after bags were resealed after each time they were opened (Magan and Aldred, 2007). This reestablishment of low oxygen concentration appeared to occur at a lower rate during the later weeks of storage, likely caused in part by decreased seed viability.

Lopez and Christensen (1967) found that *A*. *flavus* failed to invade stored maize at moisture contents below 17.5%, and Trenk and Hartman (1970) reported that aflatoxin was produced at moisture contents of 18% and higher. Both the spread of *A*. *flavus* and aflatoxin accumulation in moist maize (≥18% MC) can be controlled by placing the grain in a PICS bag (Williams et al., 2014). When the oxygen concentration reaches 5%, aflatoxin production is severely inhibited (Diener and Davis, 1977). Our results indicated that repeated opening of the PICS bags allows the aflatoxin contamination to spread to the non-inoculated maize, even at the lower 15% and 16% MC. Certainly, the oxygen levels in the lower moistures were conducive for *A*. *flavus* growth. The fact that all three replicate bags at 16% MC had measurable aflatoxin after 24 weeks indicates that the moisture of some maize kernels may have been higher than the average moisture content. Sauer and Burroughs (1980) showed that, in maize in the range of 16%–18% MC, *A*. *flavus* could grow and accumulate aflatoxin if exposed to relative humidity of 86%. Data obtained from hygrometers in the PICS bags indicated that opening the bags for 30 min at 85% RH increased pore space relative humidity.

In summary, our study examined the effects of opening PICS storage bags containing maize that was not properly dried (≤14% MC). The results indicated that grain quality, as measured by fungal growth and aflatoxin contamination, decreases over time. In practice, grain is removed from the PICS bag for consumption, thus steadily decreasing the volume of grain in the bag. Depending on the rate of consumption, our results indicated increased risk of consumer exposure to allergenic fungal spores and to aflatoxin. Therefore, it is important that the maize is properly dried prior to storage.

## Figures and Tables

**Table 1:** Effect of regular opening of hermetic bags on seed germination.^a^

Grain moisture	Initial^c^	Duration of storage^b^ (Sealed bags opened every 7 days)		Duration of storage (sealed bags - not opened) b
		8 weeks	24 weeks	24 weeks
15	97.3 ± 0.7	96.7 ± 0.4	20.8 ± 1.5	41.1 ± 4.5
16	96.0 ± 2.0	47.3 ± 3.1	1.3 ± 0.3	14.7 ± 1.9
18	98.0 ± 0.0	71.7 ± 1.6	0	0.2 ± 0.2
20	98.7 ± 1.3	6.8 ± 2.0	0	0

a Values are the means ± standard error.

b For bags opened at 8 and 24 weeks period, samples of 150 seeds were collected from the top, middle, and bottom of each replicated bag (n = 450). The sealed bags (one per moisture) were sampled after 24 weeks of storage. Samples of 150 seeds were collected from the top, middle, and bottom.

c For the initial germination, 150 seeds were tested for each moisture.

**Table 2:** Effect of regular opening of hermetic bags on aflatoxin in the non-inoculated maize.^a^

Moisture	8 weeks		24 weeks	
	# Bags with aflatoxin	Aflatoxin range	# Bags with aflatoxin	Aflatoxin range
15%	1	33	1	6.3
16%	1	20.5	3	22–115
18%	1	51.0	2	10–65
20%	1	87.7	2	7.5–12

a Initial aflatoxin B1 was <1 ng AFB1/gram of maize. Data are ng AFB1/g of maize.

## References

[bib1] Bailey S.W. (1959). The rate of diffusion of oxygen through grain. J. Sci. Food Agric..

[bib2] Baoua I.B., Amadou L., Ousmane B., Baributsa D., Murdock L.L. (2014). PICS bags for post-harvest storage of maize grain in West Africa. J. Stored Prod. Res..

[bib3] Baoua I.B., Amadou L., Murdock L.L. (2013). Triple bagging for cowpea storage in rural Niger: questions farmers ask. J. Stored Prod. Res..

[bib4] Diener U.L., Davis N.D. (1977). Aflatoxin Formation in Peanuts by *Aspergillus flavus*.

[bib5] Huang H.B., Danao M.G.C., Rausch K.D., Singh V. (2013). Diffusion and production of carbon dioxide in bulk corn at various temperatures and moisture contents. J. Stored Prod. Res..

[bib6] Lacey J. (1989). Pre-harvest and post-harvest ecology of fungi causing spoilage of foods and other stored products. J. Appl. Bacteriol..

[bib7] Lopez L.C., Christensen C.M. (1967). Effect of moisture content and temperature on invasion of stored corn by *Aspergillus flavus*. Phytopathology.

[bib8] Magan N., Aldred D. (2007). Post-harvest control strategies: minimizing mycotoxins in the food chain. Int. J. Food Microbiol..

[bib9] McDonough M.X., Campabadal C.A., Mason L.J., Maier D.E., Denvir A., Woloshuk C. (2011). Ozone application in a modified screw conveyor to treat grain for insect pests, fungal contaminants, and mycotoxins. J. Stored Prod. Res..

[bib10] Murdock L.L., Baoua I.B. (2014). On Purdue Improved Cowpea Storage (PICS) technology: background, mode of action, future prospects. J. Stored Prod. Res..

[bib11] Murdock L.L., Margam V., Baoua I., Balfe S., Shade R.E. (2012). Death by desiccation: effects of hermetic storage on cowpea bruchids. J. Stored Prod. Res..

[bib12] Mutungi C.M., Affognon H., Njoroge A.W., Baributsa D., Murdock L.L. (2014). Storage of mung bean (*Vigna radiata* [L.] Wilczek) and pigeonpea grains (*Cajanus cajan* [L.] Millsp) in hermetic triple-layer bags stops losses caused by *Callosobruchus maculatus* (F.) (Coleoptera: Bruchidae). J. Stored Prod. Res..

[bib13] Njoroge A.W., Affognon H.D., Mutungi C.M., Manono J., Lamuka P.O., Murdock L.L. (2014). Triple bag hermetic storage delivers a lethal punch to *Prostephanus truncatus* (Horn) (Coleoptera: Bostrichidae) in stored maize. J. Stored Prod. Res..

[bib14] Sauer D.B., Burroughs R. (1980). Fungal growth, aflatoxin production, and moisture equilibration in mixtures of wet and dry corn. Phytopathology.

[bib15] Shunmugam G., Jayas D., White N.D.G., Muir W.E. (2005). Diffusion of carbon dioxide through grain bulks. J. Stored Prod. Res..

[bib16] Standards A. (2012). S352.2 APR1988 (R2012) Moisture Measurement-Grain and Seeds.

[bib17] Sudini H., Rao G.V.R., Gowda C.L.L., Chandrika R., Margam V., Rathore A., Murdock L.L. (2015). Purdue Improved Crop Storage (PICS) bags for safe storage of groundnuts. J. Stored Prod. Res..

[bib18] Trenk H.L., Hartman P.A. (1970). Effects of moisture content and temperature on aflatoxin production in corn. Appl. Microbiol..

[bib19] Vales M.I., Rao G.V.R., Sudini H., Patil S.B., Murdock L.L. (2014). Effective and economic storage of pigeonpea seed in triple layer plastic bags. J. Stored Prod. Res..

[bib20] Williams S.B., Baributsa D., Woloshuk C. (2014). Assessing Purdue Improved Crop Storage (PICS) bags to mitigate fungal growth and aflatoxin contamination. J. Stored Prod. Res..

